# Role of Serum Brain‐Derived Neurotrophic Factor as a Biomarker of Chronic Pain in Older Adults

**DOI:** 10.1002/ejp.70014

**Published:** 2025-04-13

**Authors:** R. Ortolá, M. Sotos‐Prieto, A. Carballo, S. Cabello‐Plan, Aida Koni, V. Mustieles, L. M. García‐Segura, A. R. Artalejo, F. Rodríguez‐Artalejo, E. García‐Esquinas

**Affiliations:** ^1^ Department of Preventive Medicine and Public Health, School of Medicine Universidad Autónoma de Madrid Madrid Spain; ^2^ CIBER of Epidemiology and Public Health (CIBERESP) Madrid Spain; ^3^ Department of Environmental Health and Nutrition Harvard T.H. Chan School of Public Health Boston Massachusetts USA; ^4^ IMDEA Food Institute, CEI UAM+CSIC Madrid Spain; ^5^ Aging Research Center, Department of Neurobiology Care Sciences and Society Karolinska Institutet & Stockholm University Stockholm Sweden; ^6^ Universidad Nacional de Educación a Distancia (UNED) Madrid Spain; ^7^ Instituto de Investigación Biosanitaria Ibs University of Granada Granada Spain; ^8^ Center for Biomedical Research (CIBM) Granada Spain; ^9^ Instituto Cajal, CSIC Madrid Spain; ^10^ Department of Pharmacology and Toxicology, Veterinary Faculty Universidad Complutense de Madrid Spain; ^11^ National Center of Epidemiology Carlos III Health Institute Madrid Spain

## Abstract

**Background:**

Serum brain‐derived neurotrophic factor (BDNF) has emerged as a promising biomarker for chronic pain (CP) research and treatment. Yet, most human studies have been limited by small sample sizes, inadequate control of confounders and a lack of focus on sex and mental health differences.

**Methods:**

This study included data from 1932 community‐dwelling individuals aged ≥ 65 years, randomly sampled from the Spanish general population. Serum BDNF was quantified by ELISA. CP characteristics were assessed using the European Chronic Pain Survey and classified according to electronic medical records (ICPC‐2 codes). Linear regression models—adjusted for sociodemographic, lifestyle and clinical factors—and stratified analyses by sex and depression status (defined by Geriatric Depression Scale score, recent physician diagnosis or antidepressant use) were performed.

**Results:**

Among 962 men and 970 women, mean BDNF concentrations were 18.55 (5.66) ng/mL and 19.39 (5.77) ng/mL, respectively. Most participants reported pain in multiple locations (median 3 sites, interquartile range: 2–4). In 511 participants with CP, probable musculoskeletal pain was predominant (*n* = 446), followed by nociplastic (*n* = 71), neuropathic (*n* = 54), visceral (*n* = 51) and vascular pain (*n* = 22). Notably, in non‐depressed participants (*n* = 1639), women with severe or interfering pain showed lower BDNF concentrations [β coefficient (95% confidence interval) = −2.62 ng/mL (−5.03, −0.22) and −3.09 ng/mL (−4.71, −1.47), respectively] compared to those without CP—a pattern not seen in men. Conversely, among men with depression (*n* = 293), both severe [−5.12 g/mL (−9.26, −0.99)] and interfering [−4.95 g/mL (−8.29, −1.61)] pain were linked to lower BDNF, a trend absent in depressed women. Similar associations were observed in analyses of musculoskeletal and nociplastic pain subtypes.

**Conclusions:**

While serum BDNF is a promising biomarker for CP, its reliability for gauging pain severity depends on patient sex and depression status. These factors must be considered to enhance the accuracy and clinical relevance of BDNF in CP evaluation.

**Significance:**

Our study is the first to reveal that the relationship between serum BDNF and chronic pain is distinctly modulated by sex and depression. This novel insight challenges one‐size‐fits‐all biomarker approaches and paves the way for more personalised, precision‐based strategies in chronic pain diagnosis and management.

## 1 Introduction

Chronic pain (CP) is a persistent, long‐lasting pain continuing beyond the expected healing period (Raja et al. 2020). Unlike acute pain, which protects against injury, CP is maladaptive, often occurring without an obvious cause or after healing. CP triggers peripheral and central sensitisation, neural pathway changes, muscle dysfunction, hypothalamic–pituitary–adrenal axis alterations, gene expression shifts, brain structure changes and immunosuppression (Zimney et al. 2023).

Older adults are particularly vulnerable to developing CP—especially of musculoskeletal origin (International Association For the Study of Pain 2019, n.d.)—and its associated complications (Domenichiello and Ramsden 2019). Among this population, CP is strongly linked to increased risk of depression (Rodríguez‐Sánchez et al. 2019), falls (Cai et al. 2022), disability (Thakral et al. 2019), cognitive decline (Whitlock et al. 2017) and premature mortality (Macfarlane et al. 2017). Furthermore, sex and gender play critical roles in shaping the onset, progression and management of CP. Women tend to exhibit lower pain thresholds and tolerance (Greenspan et al. 2007), greater activation of brain regions involved in the emotional dimensions of pain, and higher susceptibility to disabling pain and depression due to biological, social and psychological differences (García‐Esquinas et al. 2019). These gender‐related variations may be further influenced by epigenetic mechanisms, where behaviour and environment interact with gene expression to modulate biological responses to pain (Mauvais‐Jarvis et al. 2020).

Diagnosing and managing CP remains challenging due to its reliance on subjective self‐reports, highlighting the need for objective biomarkers to improve diagnosis, monitoring and treatment. Brain‐derived neurotrophic factor (BDNF) has emerged as a promising candidate biomarker in CP research and clinical practice (Thakkar and Acevedo 2023). This neurotrophin plays a central role in neural differentiation, maturation, survival and plasticity, and it can be assessed through various methods, including DNA methylation in blood, gene expression and protein levels in urine. Among these, serum BDNF has garnered particular attention due to its practicality and relevance. It is readily measurable in significant concentrations in all individuals (Olivas‐Martinez et al. 2022), shows a strong correlation with the onset and progression of several neurodegenerative and neuropsychiatric disorders (Autry and Monteggia 2012; Sen et al. 2008) and responds to both pharmacological and nonpharmacological interventions (McGonigal et al. 2023; Meshkat et al. 2022; Pisani et al. 2023). In particular, serum BDNF is well‐studied in depression research, reflecting its potential to capture the neurobiological changes underlying depressive disorders and treatment responses, with lower circulating levels being associated with depression and anxiety (Arosio et al. 2021).

Experimental evidence from animal models links BDNF to hyperalgesia in CP (Coull et al. 2005; Xiong et al. 2024), with reported sex differences. For instance, in male mice, BDNF mediates hyperalgesia via microglial purinergic receptor signalling, whereas in females, pain‐related neuroinflammation is primarily driven by T cell activity (Paige et al. 2018; Sorge et al. 2015). Additionally, early life nerve injury in mice selectively increases BDNF in males but not in females, who exhibit more depression‐like behaviours (Nishinaka et al. 2015). Estradiol and dopamine signalling further modulate BDNF activity, potentially amplifying these sex‐specific vulnerabilities to CP‐induced emotional disorders (Deb et al. 2024). Moreover, epigenetic factors, such as sex‐specific chromatin accessibility and transcriptional regulation of nociceptive genes, may contribute to differential CP outcomes between sexes (Franco‐Enzástiga et al. 2024). While these findings provide valuable mechanistic insights in preclinical models, their direct applicability to human physiology remains to be fully established.

In human studies, elevated plasma and serum BDNF levels have been generally reported in CP patients compared to healthy controls (Behnoush et al. 2023; Sorkpor et al. 2021; Wang and Li 2021). However, human studies often have limitations, including small sample sizes (typically fewer than 100 participants) and insufficient control for confounding factors. Furthermore, sex‐specific associations between CP and BDNF in older populations remain largely unexplored, and the impact of coexisting CP and depression on BDNF is rarely considered. Considering the gaps in the literature, this study aimed to (1) identify differences in serum BDNF levels between older adults with and without CP; (2) investigate serum BDNF associations with CP severity and intensity; and (3) assess whether these associations are modified by sex or depression status.

## 2 Methods

### 2.1 Study Design and Participants

A total of 3273 participants aged 65 years or older were randomly selected from the Community of Madrid health card registry (Seniors‐Enrica‐2 cohort: ClinicalTrials.gov NCT03541135) (García‐Esquinas et al. 2021). The selection process involved sex‐ and district‐stratified random sampling among individuals holding a national healthcare card, representing approximately 99% of the population of the Spanish region. Trained personnel collected information from participants using standardised protocols at three sequential stages. First, a telephone interview covered sociodemographic factors, health behaviours, self‐rated health and chronic morbidities. Next, during a home visit, trained nurses performed physical examinations and collected blood samples. Finally, during an additional home visit, an electronic and validated dietary history was obtained (Rodríguez‐Artalejo et al. 2011). Participants also carried an ambulatory blood pressure monitor for 24 h and a wrist‐worn accelerometer for 1 week (Cabanas‐Sánchez et al. 2021). The study protocol was approved by the Clinical Research Ethics Committee of the Hospital Universitario ‘La Paz’ of Madrid (Protocol #HULP‐PI 1793), and participants gave written informed consent.

### 2.2 Study Variables

#### 2.2.1 BDNF

Fasting blood samples were obtained in rapid serum tubes with a thrombin‐based coagulation activator and polymer gel (Becton Dickinson). After centrifugation at 1000G for 10 min, the serum was aliquoted and stored at −80°C until analysis. All experimental procedures were performed at the Department of Preventive Medicine and Public Health, Universidad Autónoma de Madrid. Total serum BDNF levels were assessed using the high‐sensitivity and human‐specific BDNF Enzyme‐Linked Immunosorbent Assay (Cat. #EH0043, Fine Test; sensitivity 18.75 pg/mL) following the manufacturer's instructions. Duplicate measurements were carried out at controlled room temperature and processed by the same operator to ensure consistency. The average of both duplicate measurements was used.

Briefly, 96 well plates were coated with a standard solution (bounded to biotin‐conjugated antibodies) and streptavidin‐horseradish peroxidase (HRP) for standard solution wells. After incubation at 37°C for 60 min and subsequent washing, the plates were incubated with 3,3′,5,5′‐Tetramethylbenzidine (TMB) for 10 min at 37°C. TMB was catalysed by HRP to produce a blue colour product that changed into yellow after adding acidic stop solution. The optical density, which is proportional to the target amount of sample captured in the plate, was measured at a wavelength of 450 nm using a BioTek 800TS automated microplate reader within 10 min of adding the stop solution. Each sample was analysed in two separate wells, with serum diluted 1:40. Plates were analysed on different days in the same laboratory, with two plates processed per day. Each plate included a standard curve and 40 dilute samples to ensure accuracy. Blank samples were used to account for background noise. The BDNF concentration for each sample was determined using a standard curve generated according to the manufacturer's protocol. The inter‐assay and intra‐assay coefficients of variation (CV) were below 8% and 10%, respectively, ensuring high reliability and precision of the measurements.

#### 2.2.2 Pain

CP was defined as current pain lasting for more than 6 months and was evaluated using 10 questions from the European Chronic Pain Survey (Breivik et al. 2006), covering pain intensity and interference with daily activities. Consistent with previous studies, pain intensity was assessed using a 1‐to‐10 scale and classified as severe if rated above 7 (Rodríguez‐Sánchez et al. 2019). Pain location was reported in six categories: (a) head and neck; (b) back; (c) bones and joints; (d) legs; (e) arms; (f) abdomen; (g) chest; and (h) other sites. Pain was deemed interfering when it moderately or completely disrupted daily activities (Rodríguez‐Sánchez et al. 2019).

To complement the information on CP, we utilised electronic health records from primary care to evaluate potential causes, classified using the International Classification of Primary Care‐2 (ICPC‐2) codes. Participants with CP were categorised based on their diagnoses as follows: 1‐ Individuals with CP in locations (a) to (e) and diagnoses of muscle pain, bursitis, tendinitis, shoulder syndromes, rheumatoid arthritis (RA), ankylosing spondylitis, osteoarthritis (OA) or other degenerative joint diseases (L18, L85, L88, L88‐01, L87, L89, L90, L91, L92, L94, L98, T92) were considered to have probable musculoskeletal pain (*n* = 446); 2‐ Individuals diagnosed with migraine, cluster headache, trigeminal neuralgia, neuritis or peripheral neuropathies (codes N89, N90, N91, N92 and N94) were classified as having probable neuropathic pain (*n* = 54); 3‐ Participants with CP and diagnoses of fibromyalgia, anxiety disorders, conversion disorders, neurasthenia, phobia or post‐traumatic stress disorder (L18_01, P74, P75, P78, P79, P84) were classified as having potential nociplastic pain (*n* = 71); 4‐ Individuals with diagnoses of irritable bowel syndrome, chronic enteritis, inflammatory bowel disease or pelvic inflammatory disease (D72, D84, D85, D86, D92, D93, D94, D98, X74) were categorised as having potential visceral pain (*n* = 51); 5‐ Individuals with CP in the lower limbs and a diagnosis of peripheral arterial disease, phlebitis/thrombophlebitis, superficial or deep venous thrombosis (K92, K94, K94_01, K94_02) were classified as having probable vascular pain (*n* = 22).

### 2.3 Sociodemographic Variables, Lifestyle Behaviours, Cardiometabolic Risk Factors and Comorbid Conditions

We included in the analyses variables that could potentially confound the association between BDNF levels and CP. These variables were selected based on their established relevance in the literature and their potential to influence both BDNF levels and CP outcomes. Age, sex, education (< high school, high school and > high school), smoking (never, former and current smoker), alcohol intake (never, former, moderate and heavy drinkers) and sleep quality were self‐reported by study participants. Recreational physical activity (comprising walking, cycling and sports) was assessed with the EPIC‐Spain cohort questionnaire, while sedentary behaviour was estimated as time spent watching television using the Nurse's Health Study questionnaire validated in Spain. In a subset of participants (*n* = 2000), accelerometers were worn for 24‐h activity cycle assessment, including sleep parameters (Cabanas‐Sánchez et al. 2021). Adherence to the Mediterranean diet was measured with the Mediterranean Diet Adherence Screener (MEDAS) score.

In the home interviews, participants' body weight and height were measured, and the body mass index (BMI) was calculated as weight in kg divided by squared height in m. Blood pressure was measured thrice at 1–2‐min intervals, and we averaged the last two readings. Medication use, including antidepressants, antihypertensive, lipid‐lowering and antidiabetic drugs was self‐reported and further confirmed by checking the drug packages during home visits. Fasting blood samples were analysed for creatinine, glucose, total cholesterol, high‐density lipoprotein (HDL) cholesterol and triglycerides using enzymatic methods on an Atellica Solution analyser (Siemens Healthineers). Glomerular filtration rate (GFR) was estimated from plasma creatinine values using the Chronic Kidney Disease Epidemiology Collaboration (CKD‐EPI) equation.

Type 2 diabetes was defined by self‐reported medical diagnosis, fasting glucose ≥ 126 mg/dL or antidiabetic medication use. Depression was operationally defined by meeting at least one of the following criteria: self‐reported physician‐diagnosed depression during the previous 12 months, current use of antidepressant medication, or scoring 3 or more points on the 10‐item version of the Geriatric Depression Scale (GDS) at the time of the interview. Mental wellbeing was measured with the mental component summary (MCS) of the 12‐item Short Form Survey (SF‐12) questionnaire. Finally, physician diagnoses of chronic conditions, such as cardiovascular disease (ischemic heart disease, stroke or heart failure), chronic lung disease (asthma or chronic bronchitis), hip fracture, OA and RA, were also self‐reported.

### 2.4 Statistical Analyses

In this study, we analysed the association between serum BDNF concentrations—which followed a normal distribution—and CP characteristics using linear regression models progressively adjusted for potential confounders. Model 0 did not adjust for any potential confounders; model 1 was adjusted for sex and age; model 2 was further adjusted for educational level, lifestyle risk factors and chronic conditions; model 3 additionally adjusted for mental wellbeing (and GDS scores among participants with depression); and model 4 was further adjusted for quartiles of platelet count and mean platelet volume. We examined effect modification by sex and depression status by conducting likelihood ratio tests that compared models with and without interaction terms between BDNF concentrations and these variables. Because the results revealed that these factors significantly modified the study associations, all the analyses are presented separately for men and women and for participants with and without depression. Finally, as sensitivity analyses, we performed stratified analyses based on medically diagnosed CP conditions and conducted additional analyses restricting the sample to participants with CP.

## 3 Results

A total of 3273 participants were initially considered for this study. After applying specific exclusion criteria, the final analytical sample consisted of 1932 participants, classified into two groups based on their mental health. The group without depression (*n* = 1639) included participants with no diagnosis of depression in the previous 12 months, ≤ 2 symptoms on the GDS‐10 scale, and no use of antidepressant medications. The group with depression (*n* = 293) comprised participants who met the criteria for probable depression based on the same indicators.

**FIGURE 1:**
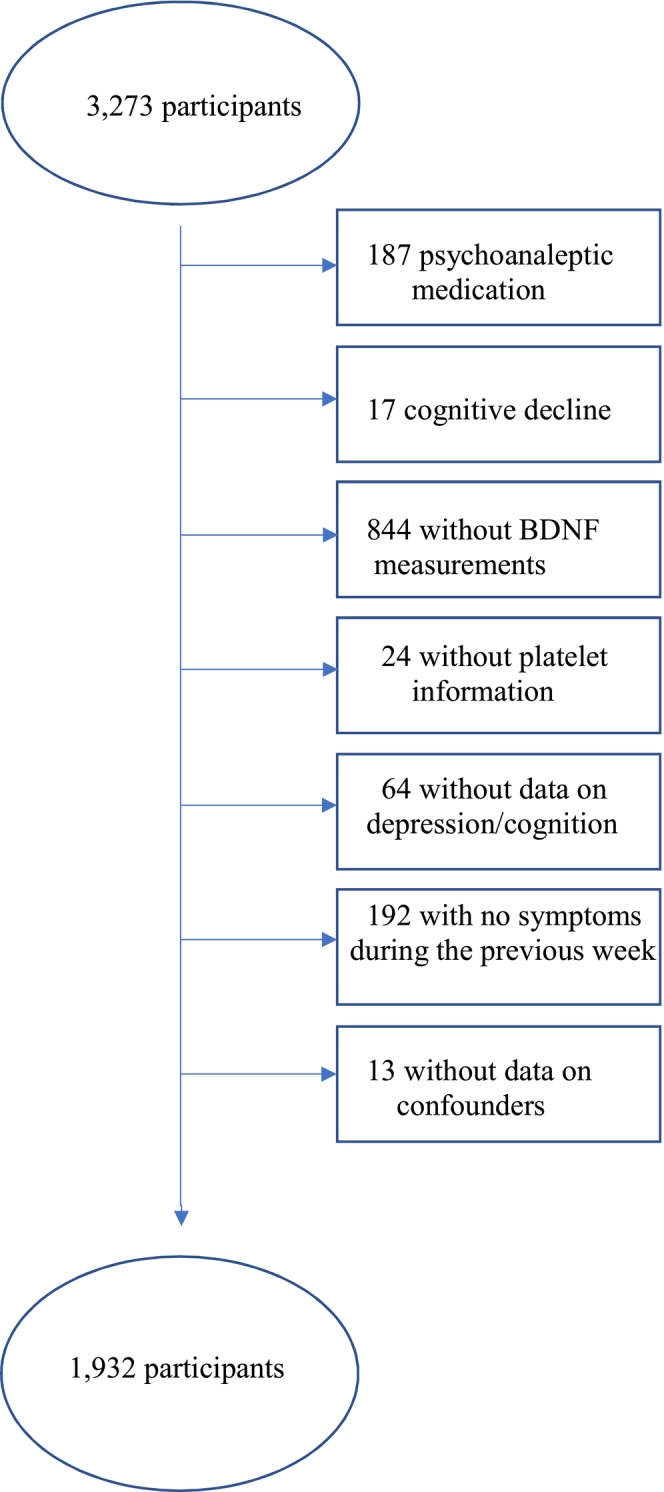
Flow chart of Participants Included and Excluded in the Analyses.

Participants were excluded for the following reasons: (1) use of psychoanaleptic drugs (ATC N06 classification, *n* = 187), as these medications can alter peripheral BDNF concentrations (Merabtine et al. 2024); (2) presence of moderate to severe cognitive decline (MMSE ≤ 20, *n* = 17); (3) lack of blood samples due to absence of home visits (*n* = 801); (4) missing BDNF determinations despite blood sample collection (*n* = 43); (5) missing platelet count or mean platelet volume data (*n* = 24), given the critical role platelets play in storing and modulating circulating BDNF concentrations (Naegelin et al. 2018), and their association with psychological and pain‐related syndromes (Fábián et al. 2022; Haliloğlu et al. 2014); (6) lack of information on depression or cognitive decline (*n* = 64); (7) reporting CP but no symptoms in the last week (*n* = 192); and (8) insufficient data on potential confounders (*n* = 13). The detailed exclusion process is summarised in Figure 1.

The final sample consisted of 962 men and 970 women, with an average of 71 years. Descriptive statistics revealed that women exhibited a higher prevalence of CP (34.5% vs. 18.3%) and depression (22.1% vs. 8.2%) compared to men. Among participants with CP (*n* = 511), the prevalence of depression was significantly higher at 31.1%, compared to 9.4% among those without CP. Most participants reported experiencing pain in multiple locations, with a median of 3 pain sites (interquartile range: 2–4). Regarding pain type, 446 participants were classified as having probable musculoskeletal pain, either in isolation (83.9%) or combined with one or more additional types of pain. Additionally, 71 participants were classified as having potential nociplastic pain, 54 as having probable neuropathic pain, 51 as having potential visceral pain and 22 as having probable vascular pain.

Serum BDNF concentrations were evaluated across various participant characteristics to explore baseline differences. Among individuals without depression, women showed slightly higher serum BDNF levels than men. Higher BDNF concentrations were also observed in current smokers, former drinkers and those in the highest quartile of platelet count and lowest quintile of platelet volume. Although slight variations in BDNF were noted across self‐reported and accelerometer‐based PA tertiles, these differences were not statistically significant. Generally, participants without chronic conditions (e.g., hypertension, cardiovascular disease, cancer, diabetes and chronic respiratory disease) displayed slightly higher BDNF concentrations. These patterns, further detailed in Table 1, were consistent in the group with depression (Table S1) and provided important context for understanding potential confounders and set the stage for subsequent analyses addressing the relationship between BDNF and CP.

**TABLE 1:** Mean (SD) concentrations of serum BDNF (ng/mL) in not‐cognitively impaired older adults without depression by participant's characteristics, stratified by sex (*n* = 1639).

	*n*	Men *n* = 883	*n*	Women *n* = 756
		Mean (SD)		Mean (SD)
Overall		18.5 (5.69)		19.3 (5.83)
Age (years)				
< 70	364	18.3 (5.4)	276	19.4 (5.2)
70–75	331	18.8 (5.5)	284	19.2 (5.9)
> 75	188	18.2 (6.6)	196	19.1 (6.5)
Educational level				
< High school	468	18.6 (6.0)	517	19.0 (5.7)
High school	195	18.7 (5.7)	135	20.3 (8.2)
> High school	229	18.0 (5.0)	104	19.5 (5.9)
Smoking				
Never	274	18.9 (5.7)	532	18.9 (5.8)
Former	513	18.1 (5.6)	179	20.0 (5.5)
Current	96	19.5 (5.7)	45	20.4 (6.7)
Alcohol drinking				
Never drinker	50	20.2 (5.9)	210	19.0 (6.2)
Moderate drinker	704	18.3 (5.7)	490	19.3 (5.7)
Heavy drinker	74	18.4 (4.9)	22	18.7 (3.6)
Former drinker	49	19.0 (6.2)	31	21.6 (4.9)
MEDAS				
Tertile 1	275	18.5 (5.9)	258	19.4 (5.4)
Tertile 2	396	18.5 (5.4)	323	19.3 (6.2)
Tertile 3	212	18.4 (6.0)	175	19.1 (5.6)
Self‐reported recreational PA				
Tertile 1	274	18.8 (5.7)	289	19.7 (6.3)
Tertile 2	302	18.0 (5.9)	199	18.8 (5.3)
Tertile 3	307	18.6 (5.5)	268	19.2 (5.6)
Self‐reported TV viewing time				
Tertile 1	303	18.8 (6.1)	267	19.4 (6.1)
Tertile 2	295	18.0 (5.7)	359	19.3 (5.6)
Tertile 3	285	18.6 (5.3)	130	19.0 (5.8)
Accelerometer‐based PA^a^				
Tertile 1	266	18.7 (5.4)	232	18.3 (5.5)
Tertile 2	285	18.3 (5.5)	246	18.4 (6.0)
Tertile 3	279	18.4 (6.0)	246	19.4 (5.9)
Accelerometer‐based sedentary time^a^				
Tertile 1	278	18.3 (6.1)	242	19.4 (5.9)
Tertile 2	287	18.7 (5.4)	245	19.2 (5.4)
Tertile 3	265	18.4 (5.5)	237	19.3 (6.2)
Body Mass Index (kg/m^2^)				
< 25	208	19.0 (5.3)	247	19.4 (5.6)
25 to < 30	475	18.2 (5.7)	327	19.3 (5.9)
≥ 30	200	18.6 (6.0)	183	19.2 (6.0)
Sleep time				
Tertile 1	280	18.7 (6.0)	247	19.4 (6.0)
Tertile 2	281	18.1 (5.3)	243	19.3 (5.6)
Tertile 3	269	18.5 (5.6)	234	19.1 (5.9)
Hypertension				
No	269	18.3 (5.8)	263	19.5 (6.0)
Yes	614	18.6 (5.6)	493	19.2 (5.8)
Cardiovascular disease				
No	860	18.5 (5.7)	737	19.3 (5.8)
Yes	23	17.6 (5.1)	19	19.5 (6.6)
Cancer				
No	854	18.5 (5.7)	741	19.3 (5.8)
Yes	29	18.2 (5.2)	15	19.8 (5.4)
Diabetes				
No	675	18.4 (5.7)	642	19.2 (5.8)
Yes	208	18.5 (5.7)	114	19.7 (6.0)
Rheumatoid arthritis				
No	857	18.4 (5.7)	699	19.2 (5.8)
Yes	26	20.7 (6.5)	57	19.7 (5.8)
Osteoarthritis				
No	646	18.4 (5.7)	354	19.3 (5.6)
Yes	237	18.7 (5.6)	402	19.2 (6.1)
Chronic respiratory disease				
No	837	18.4 (5.7)	691	19.2 (5.9)
Yes	46	19.3 (4.9)	65	19.7 (5.6)
Mental component of the SF12				
Tertile 1	296	18.2 (5.9)	178	19.1 (6.3)
Tertile 2	316	18.8 (5.5)	286	19.7 (6.2)
Tertile 3	271	19.3 (5.7)	292	18.9 (5.1)
Platelet count (×10^9^/L)				
Quartile 1	317	16.7 (5.4)	122	16.8 (4.9)
Quartile 2	222	18.3 (5.3)	180	18.7 (5.6)
Quartile 3	191	19.5 (5.5)	209	19.6 (5.7)
Quartile 4	153	20.9 (6.0)	245	20.6 (6.1)
Platelet volume				
Quartile 1	217	19.4 (6.2)	199	19.5 (6.5)
Quartile 2	206	18.4 (5.8)	207	20.0 (5.6)
Quartile 3	229	17.9 (5.7)	183	18.5 (5.3)
Quartile 4	231	18.2 (5.1)	167	18.9 (5.7)

Abbreviations: MEDAS, Mediterranean Diet Adherence Screener; PA, physical activity.

^a^ Information available in the subset of participants who wore an accelerometer.

**FIGURE 2:**
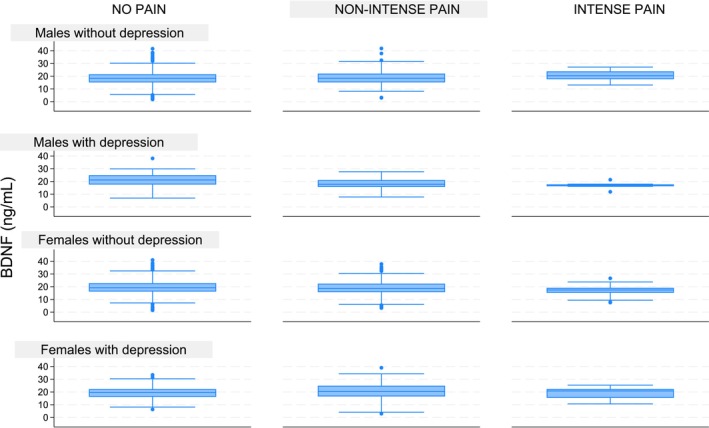
Distribution of BDNF Levels Stratified by Sex and Depression Status.

The crude relationship between BDNF concentrations and pain intensity was analysed by stratifying the sample by sex and depressive status. Results showed that mean BDNF concentrations were slightly higher in participants with depression (19.7 ng/mL ± 5.5) compared to those without (18.8 ng/mL ± 5.8). However, among men with CP and depression, BDNF concentrations were lower (17.9 ng/mL ± 3.9) compared to their counterparts without depression (19.2 ng/mL ± 6.0). Additionally, in men—but not women—with CP, BDNF concentrations decreased with increasing depressive symptom severity, as measured by GDS scores. Specifically, for each one‐point increase in GDS, mean BDNF levels decreased by −0.60 ng/mL (95% CI: −1.05, −0.15) in men, while no significant association was observed in women (−0.12 ng/mL; 95% CI: −0.61, 0.36). These findings, illustrated in Figure 2, help to clarify the interaction between depression severity and BDNF levels in the context of CP.

To isolate the effect of pain on BDNF levels while controlling for other potential confounders, multivariate analyses were performed, which further highlighted sex‐specific differences (Table 2). Among individuals without depression, results showed that, compared to women without pain, those with severe or interfering pain showed mean differences (MD) in BDNF of −2.62 ng/mL (95% CI: −5.03, −0.22) and −3.09 ng/mL (95% CI: −4.71, −1.47), respectively. A one‐point increase on the VAS scale was associated with a mean decrease of −0.45 ng/mL (95% CI: −0.89, −0.01) (data not shown in tables). In contrast, no significant association was observed in men without depression, where a one‐point increase on the VAS scale was linked to a mean increase of 0.41 ng/mL (95% CI −0.14, 0.97). However, among men with CP and depression, BDNF levels were significantly lower compared to those without CP, after fully adjusting for the number of depressive symptoms and MCS scores (Table 3). This reduction was particularly pronounced among men experiencing severe pain [MD: −5.12 ng/mL (95% CI: −9.26; −0.99)] or interfering pain [MD: −4.95 ng/mL (95% CI: −8.29; −1.61)], with similar trends in men with non‐severe or non‐interfering pain. Overall, in men without depression, a one‐point increase on the VAS scale was associated with a mean decrease of −1.53 ng/mL (95% CI: −2.67, −0.38) in this group.

**TABLE 2:** Mean differences in BDNF levels (ng/mL) by pain status and its characteristics in older adults without depression, stratified by sex (*n* = 1639).

	*n*	Model 0	Model 1	Model 2	Model 3	Model 4
Pain (vs. no pain)						
Men	137	0.89 (−0.16; 1.94)	0.83 (−0.21; 1.87)	0.82 (−0.24; 1.89)	0.90 (−0.16; 1.97)	0.76 (−0.28; 1.80)
Women	215	−0.68 (−1.59; 0.13)	−0.81 (−1.71; 0.10)	−0.97 (−1.90; −0.04)*	−0.96 (−1.89; −0.02)*	−0.91 (−1.81; −0.00)*
*p*‐int		0.027*	0.020*	0.012*	0.001*	0.016*
Intensity (VAS)						
Men						
Non‐severe (≤ 7)	121	0.71 (−0.39; 1.82)	0.64 (−0.46; 1.74)	0.64 (−0.48; 1.75)	0.71 (−0.41; 1.83)	0.67 (−0.42; 1.76)
Severe (> 7)	16	2.24 (−0.61; 5.09)	2.29 (−0.55; 5.13)	2.35 (−0.51; 5.22)	2.54 (−0.33; 5.42)	1.57 (−1.23; 4.37)
Women						
Non‐severe (≤ 7)	193	−0.49 (−1.43; 0.46)	−0.64 (−1.58; 0.30)	−0.78 (−1.75; 0.19)	−0.76 (−1.73; 0.21)	−0.71 (−1.65; 0.23)
Severe (> 7)	22	−2.35 (−4.80; 0.10)	−2.24 (−4.69; 0.20)*	−2.59 (−5.07; −0.11)	−2.67 (−5.14; −0.19)*	−2.62 (−5.03; −0.22)*
*p*‐int		0.023*	0.018*	0.009*	0.006*	0.019*
Interference in daily living						
Men						
Non‐interfering	110	1.09 (−0.06; 2.24)	1.01 (−0.13; 2.16)	0.99 (−0.17; 2.15)	1.05 (−0.12; 2.21)	0.94 (−0.20;2.07)*
Interfering	27	0.09 (−2.12; 2.29)	0.08 (−2.11; 2.28)	0.02 (−2.20; 2.24)	0.14 (−2.09; 2.37)	−0.11 (−2.28;2.05)
Women						
Non‐interfering	162	−0.01 (−1.00; 1.02)	−0.15 (−1.15; 0.86)	−0.28 (−1.31; 0.74)	−0.29 (−1.31; 0.73)	−0.25 (−1.24; 0.74)
Interfering	53	−2.78 (−4.40; −1.16)	−2.83 (−4.45; −1.21)*	−3.23 (−4.90; −1.57)*	−3.18 (−4.84; −1.51)*	−3.09 (−4.71; −1.47)*
*p*‐int		0.056	0.048	0.025*	0.020*	0.037*

*Note:* *p*‐int: *p* value for interaction obtained from Wald tests for interaction terms. Model 0: Unadjusted. Model 1: Adjusted for age and educational level. Model 2: model 1 + further adjustment for lifestyle risk factors [i.e., tobacco smoke (never, former and current smoker); alcohol consumption (never, moderate, heavy and former drinker); recreational physical activity (METS‐h/week); television‐viewing time (hours/week); sleep quality and sleep duration; diet quality (Mediterranean Diet Adherence Score); body mass index (kg/m^2^)]; and chronic conditions (hypertension, cardiovascular disease, diabetes, cancer, osteoarthritis, rheumatoid arthritis and respiratory disease). Model 3: Model 2 + further adjusted for the mental component summary (MCS) of the 12‐item Short Form Survey (SF‐12) questionnaire. Model 4: Model 3 + further adjusted for platelet count and mean platelet volume. * *p*‐values < 0.05.

**TABLE 3:** Mean differences in BDNF levels (ng/mL) by pain status and its characteristics in older adults with depression, stratified by sex (*n* = 293).

	*n*	Model 0	Model 1	Model 2	Model 3	Model 4
Pain (vs. no pain)						
Men	39	−3.08 (−5.48; −0.68)*	−3.19 (−5.59; −0.78)*	−3.41 (−6.22; −0.60)*	−3.64 (−6.40; −0.87)*	−3.43 (−5.98; −0.88)*
Women	120	0.77 (−0.70; 2.24)	0.81 (−5.59; −0.78)	0.79 (−0.87; 2.45)	0.62 (−1.02; 2.26)	0.79 (−0.81; 2.21)
*p*‐int		0.008*	0.005*	0.006*	0.005*	0.003*
Intensity (VAS)						
Men						
Non‐severe (≤ 7)	31	−2.84 (−5.40; −0.29)	−2.94 (−5.50; −0.39)*	−3.16 (−6.12; −0.20)*	−3.27 (−6.18; −0.36)*	−3.09 (−5.77; −0.42)*
Severe (> 7)	8	−3.98 (−8.11; −0.02)	−4.10 (−8.25; −0.04)*	−4.77 (−9.33; −0.21)*	−5.49 (−10.0; −0.98)*	−5.12 (−9.26; −0.99)*
Women						
Non‐severe (≤ 7)	98	1.04 (−0.50; 2.58)	1.05 (−0.51; 2.61)	0.93 (−0.79; 2.65)	0.77 (−0.93; 2.46)	0.86 (−0.71; 2.42)
Severe (> 7)	22	−0.42 (−2.94; 2.11)	−0.21 (−2.74; 2.31)	−0.00 (−2.82; 2.81)	−0.19 (−2.97; 2.58)	−0.16 (2.70; 2.38)
*p*‐int		0.029*	0.022*	0.022*	0.161*	0.011
Interference						
Men						
Non‐interfering	24	−2.68 (−5.44; 0.07)	−2.66 (−5.42; 0.10)	−2.77 (−5.90; 0.36)	−2.81 (−5.89; 0.27)	−2.66 (−5.48; 0.17)
Interfering	15	−3.71 (−6.94; −0.48)	−4.04 (−7.29; −0.80)*	−4.66 (−8.33; −0.99)*	−5.26 (−8.89; −1.63)*	−4.95 (−8.29; −1.61)*
Women						
Non‐interfering	71	1.16 (−0.52; 2.84)	1.13 (−0.57; 2.82)	1.06 (−0.79; 2.90)	0.92 (−0.89; 2.74)	1.02 (−0.66; 2.70)
Interfering	49	0.21 (−1.67; 2.09)	0.35 (−1.55; 2.24)	0.23 (−1.91; 2.38)	−0.05 (−2.17; 2.07)	0.01 (−1.94; 1.95)
*p*‐int		0.027*	0.019*	0.019*	0.014*	0.009

*Note:* *p*‐int: *p* value for interaction obtained from Wald tests for interaction terms. Model 0: Unadjusted. Model 1: Adjusted for age and educational level. Model 2: model 1 + further adjustment for lifestyle risk factors [i.e., tobacco smoke (never, former and current smoker); alcohol consumption (never, moderate, heavy and former drinker); recreational physical activity (METS‐h/week); television‐viewing time (hours/week); sleep quality and sleep duration; diet quality (Mediterranean Diet Adherence Score; body mass index (kg/m^2^))] and chronic conditions (hypertension, cardiovascular disease, diabetes, cancer, osteoarthritis, rheumatoid arthritis and respiratory disease) Model 3: Model 2 + further adjusted for the mental component summary (MCS) of the 12‐item Short Form Survey (SF‐12) questionnaire and for the Geriatric Depression Scale (GDS) scores. Model 4: Model 3 + further adjusted for platelet count and mean platelet volume. * *p*‐values < 0.05.

Further analyses examined associations between different pain types and BDNF levels, as detailed in Table S2. Since most participants with CP reported pain of probable musculoskeletal origin, findings for the overall sample aligned with those for musculoskeletal pain. Consistency in findings was also observed for other types of pain, despite a smaller sample size, although participants with probable neuropathic and vascular pain exhibited decreased BDNF concentrations across the board. In women, the most significant reductions in BDNF levels associated with CP were observed in those with probable nociplastic and vascular pain [MD: −2.72 and −2.86 for non‐depressed and depressed women, respectively]. Among men, the most pronounced decreases in BDNF concentrations were found in those with depression and nociplastic pain (−7.02), followed by neuropathic (−5.71) and visceral pain (−5.66) in descending order. Analysing the data by pain type allowed us to identify whether specific pain mechanisms are more strongly associated with altered BDNF levels, thus contributing to a more nuanced understanding of the biological underpinnings of CP.

Restricted analyses were conducted within the subgroup of participants with CP (Table S3) to further evaluate the associations between pain characteristics and BDNF levels. In women without depression, those with severe and interfering pain showed mean BDNF reductions of −1.72 (−4.43, 0.98) and −2.39 (−4.32, −0.45), respectively, compared to those with non‐severe and non‐interfering pain. These associations were not observed in men (*p*‐interactions = 0.090 and 0.016, respectively). Among individuals with depression, no significant differences in BDNF levels were observed between those with severe versus non‐severe pain or interfering versus non‐interfering pain. This additional step helped us to pinpoint differences within a more homogeneous group of CP sufferers, thereby refining our understanding of how pain severity and interference might differentially affect BDNF levels depending on sex and depression status.

## 4 Discussion

In this cohort of community‐dwelling older adults, we identified distinct sex‐specific patterns in the relationship between CP and BDNF concentrations. Women with CP but without depression exhibited lower BDNF concentrations compared to their counterparts without CP, while men displayed an opposite trend. Among men experiencing CP alongside depression, BDNF concentrations were also significantly diminished compared to those without pain, particularly in cases of severe or interfering pain. These findings suggest that the interplay between pain and depression—especially in men—may influence reductions in BDNF concentrations, potentially reflecting the neurobiological consequences of these conditions. Our results further indicate that the decline in BDNF among CP patients is not contingent on the specific type of pain experienced. However, the overlap of multiple pain types, particularly musculoskeletal pain, complicates the interpretation of these associations. This complexity underscores the multifaceted nature of CP in older adults and underscores the need for more precise classification of pain subtypes in future research.

The relationship between CP and circulating BDNF levels has been investigated in approximately thirty human studies (Behnoush et al. 2023; Blandini et al. 2006; Dimmek et al. 2021; Holmuratova et al. 2023; Kosciuczuk et al. 2022; Mozafarihashjin et al. 2022; Wang and Li 2021; Yang et al. 2022), with most focusing on fibromyalgia (Behnoush et al. 2023). However, many of these studies are limited by small sample sizes (typically fewer than 100 participants), a focus on a single sex—commonly female for fibromyalgia and male for other pain conditions—and inadequate control of potential confounders or effect modifiers, such as physical activity, depression or antidepressant use. Furthermore, most investigations do not include people over 60 years and primarily focus on conditions like chronic widespread pain and chronic primary headache pain, limiting their generalisability and cross‐condition comparability. Even among these more extensively researched conditions, the findings remain inconsistent. For instance, research on migraine pain has yielded mixed results, with some studies reporting lower serum BDNF levels during acute and chronic migraine (Blandini et al. 2006; Mozafarihashjin et al. 2022), while others show higher BDNF levels during headache attacks compared to attack‐free periods (Holmuratova et al. 2023). In fibromyalgia, clinical studies that exclusively enrol women tend to report elevated BDNF levels (Behnoush et al. 2023) and find positive correlations between serum BDNF levels, increased motor cortex excitability disinhibition and enhanced function of the descending pain modulation system in affected women (Caumo et al. 2016) However, studies that include both men and women more often have observed a lack of elevated BDNF levels in fibromyalgia patients (Baumeister et al. 2019; Dimmek et al. 2021) suggesting potential sex differences in BDNF regulation. Unfortunately, given the small sample size of participants with CP and migraine (*n* = 28) or fibromyalgia (*n* = 31), we were unable to thoroughly evaluate the impact of CP on BDNF in the context of these conditions.

Research on other pain types remains limited. For example, one study identified a link between higher serum BDNF concentrations and pain intensity in diabetic neuropathic pain (Wang and Li 2021). Opioid treatments for non‐cancer pain have also been shown to reduce peripheral BDNF levels (Kosciuczuk et al. 2022), with levels increasing during opioid withdrawal—a phenomenon suggestive of central pain re‐sensitisation (Zhang et al. 2016). Focusing on chronic musculoskeletal pain, a study involving 147 participants—107 women and 40 men—with OA of the hip or knee and chronic back pain reported significantly lower levels of both free and total BDNF in patients compared to healthy controls (Dimmek et al. 2021). Other studies on musculoskeletal conditions align with our findings in women and in men with depression. For instance, a study involving 31 men and 18 women reported lower plasma BDNF concentrations in patients with spondyloarthropathy (*n* = 15), RA (*n* = 15) and OA (*n* = 10), compared to healthy subjects (*n* = 9) (Rihl et al. 2005). Similarly, Caumo et al. reported decreased BDNF levels in women with musculoskeletal pain and OA (*n* = 27) compared to healthy controls (Caumo et al. 2016). Conversely, two studies in older adults, one with 17 females and 10 males, and the other with 20 females and 18 males, found increased plasma BDNF levels in knee OA patients, correlating with self‐reported pain in the acute stage of the inflammatory stages (Simão et al. 2014), and with heat pain threshold as well as the numeric rating scale of pain (Sorkpor et al. 2021). Moreover, a study of 20 RA patients found elevated levels of the pro‐BDNF/p75NTR signalling pathway levels, correlating with disease activity and contributing to inflammation and pain (Yang et al. 2022). In a mouse model, blocking this pathway reduced inflammation and pain, suggesting it as a potential therapeutic target for RA (Yang et al. 2022). Together, these findings highlight the complex relationship between chronic musculoskeletal pain and BDNF regulation, underscoring the need for further studies evaluating different CP types.

Experimental studies have highlighted how sex differences in BDNF levels contribute to variations in pain perception (Stefani et al. 2012) and the development of pain‐induced mental disorders, such as depression and anxiety (Shen et al. 2023). For instance, in the peripheral spinal cord, BDNF affects excitatory signalling differently between sexes: it increases excitatory markers and modulates NMDA glutamate receptor activity in males but has minimal effects on these pathways in females (Dedek et al. 2022). At the microglial level, BDNF signalling also exhibits sex‐specific differences: microglial purinergic receptor P2X3/4‐BDNF signalling appears essential for hyperalgesia in male mice (Paige et al. 2018; Sorge et al. 2015), whereas in females, pain‐related neuroinflammation is primarily driven by T cells (Casale et al. 2021). These distinct mechanisms may help explain why females with CP are at higher risk of depression (Prowse and Hayley 2021). Furthermore, increased dopamine D1–D2 receptor heteromer expression in females may alter BDNF–TrkB signalling, increasing vulnerability to CP‐induced depressive, anxious and socially withdrawn behaviours (Shen et al. 2023). Supporting this, animal studies show that BDNF deficiency exacerbates anxiety and depression‐like behaviours in females exposed to chronic unpredictable stress (Autry et al. 2009). Moreover, early life nerve injury selectively increases BDNF in males but not females, despite females displaying more depression‐like behaviours (Nishinaka et al. 2015). Similarly, in a hyperalgesic‐priming model, the genetic deletion of BDNF from sensory neurons prevents hyperalgesia exclusively in males (Sikandar et al. 2018). Pharmacological studies have also revealed sex differences in brain BDNF expression: NMDA receptor antagonists like dizocilpine increase cortical BDNF in female mice (Matsuki et al. 2001), while phencyclidine decreases BDNF in various brain regions of female rats (Snigdha et al. 2011). Estradiol further modulates BDNF expression and function, partially accounting for sexually dimorphic effects (Deb et al. 2024; Jorgensen and Wang 2020). However, even after ovariectomy, sex differences persist, indicating that estradiol alone cannot fully explain these variations, especially in postmenopausal women. In peripheral pain signalling, muscle macrophage‐derived IL‐1β induces hyperalgesia by promoting BDNF release from sensory neurons. Blocking IL‐1β prevents hyperalgesia, but inhibiting BDNF or its receptor TrkB is effective only in males (Hayashi et al. 2024). Interestingly, fatigue metabolites combined with IL‐1β upregulate BDNF in both sexes but contribute to activity‐induced pain solely in males (Hayashi et al. 2024). Epigenetic regulation may further explain these differences. Sex‐specific epigenetic modifications in macrophages modulate inflammation and pain signalling through p75NTR (Dourson et al. 2023). Neonatal injury reshapes the epigenetic landscape, creating persistent pain memories that last into adulthood only in females (Dourson et al. 2023). Furthermore, prenatal stress followed by chronic stress in adulthood induces sex‐specific epigenetic changes in the brain and spinal cord of rats, altering BDNF expression and amplifying visceral pain in females (Luoni et al. 2016). Recent studies also show differences in chromatin accessibility linked to varying transcriptional responses of nociceptive genes in male and female dorsal root ganglia, with BDNF and its regulatory IncRNA, BDNF AS, being affected (Franco‐Enzástiga et al. 2024). Collectively, these findings highlight the complex interplay between BDNF, sex hormones, immune signalling and epigenetic regulation, supporting the need for CP and BDNF studies to evaluate potential sex‐modifying effects and mental health in this relationship.

This study is the largest to date examining the association between CP and circulating BDNF levels. A key strength lies in our rigorous adjustment of models for various factors influencing BDNF concentrations and CP, including sex, mental health, physical activity, alcohol intake and platelet count. However, we lacked genetic information, which is significant since BDNF polymorphisms can affect mental health (Hong et al. 2011) and pain perception (Xiong et al. 2024). For instance, human studies suggest that carriers of the Val66Met gene polymorphism may exhibit differential susceptibility to major depressive disorders, altered pain perception and increased catastrophising (da Silveira Alves et al. 2020; Tian et al. 2018; Wang et al. 2023). Additionally, the Met/Met genotype of BDNF rs6265 has been linked to decreased plasma BDNF levels and heightened anxiety in panic disorder, as well as more severe cancer‐related neuropathic pain in survivors (Chu et al. 2022; Goto et al. 2023). Another limitation is the small number of participants with depression, specifically those experiencing severe or interfering pain, which diminishes the statistical power of the analyses. While evidence indicates that serum total BDNF correlates with central nervous system BDNF (Dawood et al. 2007; Karege et al. 2002), variations in serum levels may reflect adaptations in megakaryocytes and platelets due to underlying disease (Chacón‐Fernández et al. 2016). Moreover, given the differing roles of pro‐BDNF and mature BDNF isoforms on the nervous system (Li et al. 2023; Luo et al. 2016)—were pro‐BDNF preferentially binds to the p75 neurotrophin receptor and induces apoptosis, while mature BDNF activates tyrosine kinase receptors to promote cell survival and synaptic plasticity—future studies should consider exploring these isoforms for clearer conclusions. Finally, we also acknowledge that access to more specific coding systems, such as ICD‐11, would enhance understanding of the distinct mechanisms underlying various pain types. Nevertheless, the CIAP codes utilised in our study represent a clinically relevant approach for categorising CP in primary care settings, where specialised approaches are not available.

Overall, our findings suggest that serum total BDNF levels may serve as a useful biomarker for the diagnosis and management of CP, particularly in women without depressive symptoms. However, relying solely on BDNF to assess pain intensity may be unreliable without considering factors like sex and coexistence of depressive symptoms. Considering the high burden of CP and the limitations of current pharmaceutical treatments for CP in older adults, targeting BDNF therapeutically holds promise. Further research into complementary BDNF biomarkers is essential to better understand the interplay between sex, mental health and serum BDNF levels in CP, ultimately enabling more effective and personalised pain management strategies.

## Author Contributions

The authors' responsibilities were as follows – E.G.‐E.: conceptualised the study, was responsible for statistical analyses, drafted the manuscript and had primary responsibility for final content; R.O., M.S.‐P., F.R.‐A. and E.G.‐E. acquired funding and provided epidemiological data; all authors contributed to interpretation of results, reviewed the manuscript for important intellectual content, and read and approved the final manuscript.

## Supporting information


Data S1.
