# Supplementary material for: Role of Serum Brain‐Derived Neurotrophic Factor as a Biomarker of Chronic Pain in Older Adults
Source: Eur J Pain. 2025 Apr 13;29(5):e70014. doi: 10.1002/ejp.70014 (PMC11994381; doi:10.1002/ejp.70014)
Supplement: Supplementary file 1 — Data S1. [file EJP-29-0-s001.docx]

**Supplementary Table 1:**Mean (SD) Concentrations of Serum BDNF (ng/mL) in Not-Cognitively Impaired Older Adults with Depression by Participant´s Characteristics, Stratified by Sex (n=293).

|  |  | **Men**  n=79 | | | | **Women**  n=214 | | | |
| --- | --- | --- | --- | --- | --- | --- | --- | --- | --- |
|  | n |  |  | |  | n | |  |  |
| **GDS-10 (mean, SD)** |  |  | 4.00 (2.43) | |  |  | | 3.53 (2.24) |  |
| **Overall** |  |  | 19.6 (5.2) | |  |  | | 19.8 (5.66) |  |
| **Age (years)** |  |  |  | |  |  | |  |  |
| <70 | 34 |  | 19.1 (3.9) | |  | 73 | | 19.1 (4.6) |  |
| 70-75 | 26 |  | 19.8 (7.0) | |  | 87 | | 19.8 (5.5) |  |
| >75 | 19 |  | 19.7 (4.5) | |  | 54 | | 20.5 (6.7) |  |
| **Educational level** |  |  |  | |  |  | |  |  |
| <High School | 45 |  | 20.8 (5.3) | |  | 162 | | 19.9 (5.6) |  |
| High School | 17 |  | 18.0 (5.5) | |  | 30 | | 20.1 (5.1) |  |
| >High School | 17 |  | 19.0 (4.8) | |  | 22 | | 18.7 (4.0) |  |
| **Smoking** |  |  |  | |  |  | |  |  |
| Never | 26 |  | 19.4 (5.7) | |  | 155 | | 19.5 (5.7) |  |
| Former | 39 |  | 19.4 (5.5) | |  | 40 | | 20.2 (5.3) |  |
| Current | 14 |  | 19.8 (3.3) | |  | 19 | | 21.0 (5.1) |  |
| **Alcohol drinking** |  |  |  | |  |  | |  |  |
| Never drinker | 6 |  | 21.5 (4.9) | |  | 66 | | 20.9 (5.5) |  |
| Moderate drinker | 52 |  | 19.6 (5.4) | |  | 133 | | 19.1 (5.6) |  |
| Heavy drinker | 8 |  | 18.2 (4.2) | |  | 2 | | 20.5 (1.9) |  |
| Former drinker | 12 |  | 18.8 (5.8) | |  | 12 | | 20.8 (6.2) |  |
| **MEDAS** |  |  |  | |  |  | |  |  |
| Tertile 1 | 32 |  | 19.7 (5.5) | |  | 87 | | 20.0 (6.0) |  |
| Tertile 2 | 34 |  | 20.2 (5.0) | |  | 97 | | 19.8 (5.6) |  |
| Tertile 3 | 13 |  | 16.9 (4.4) | |  | 30 | | 19.9 (3.6) |  |
| **Self-reported recreational PA** |  |  |  | |  |  | |  |  |
| Tertile 1 | 48 |  | 19.1 (5.7) | |  | 117 | | 19.6 (6.1) |  |
| Tertile 2 | 18 |  | 20.3 (4.3) | |  | 43 | | 19.9 (5.6) |  |
| Tertile 3 | 13 |  | 19.5 (4.4) | |  | 54 | | 20.1 (4.1) |  |
| **Self-reported TV viewing time** |  |  |  | |  |  | |  |  |
| Tertile 1 | 25 |  | 20.0 (5.9) | |  | 59 | | 19.6 (6.1) |  |
| Tertile 2 | 19 |  | 20.5 (5.4) | |  | 100 | | 20.2 (5.1) |  |
| Tertile 3 | 35 |  | 18.5 (4.5) | |  | 55 | | 19.2 (5.8) |  |
| **Accelerometer-based PA^+^** |  |  |  | |  |  | |  |  |
| Tertile 1 | 37 |  | 19.7 (4.8) | |  | 77 | | 19.0 (6.0) |  |
| Tertile 2 | 18 |  | 19.6 (4.8) | |  | 62 | | 20.1 (5.8) |  |
| Tertile 3 | 23 |  | 19.1 (6.4) | |  | 62 | | 20.1 (5.0) |  |
| **Accelerometer-based sedentary time^+^** |  |  |  | |  |  | |  |  |
| Tertile 1 | 25 |  | 18.5 (5.0) | |  | 67 | | 20.1 (5.8) |  |
| Tertile 2 | 16 |  | 21.0 (7.5) | |  | 63 | | 20.1 (6.0) |  |
| Tertile 3 | 37 |  | 19.5 (4.1) | |  | 71 | | 19.1 (5.2) |  |
| **Body Mass Index (kg/m^2^)** |  |  |  | |  |  | |  |  |
| <25 | 18 |  | 20.8 (6.98) | |  | 60 | | 19.3 (5.3) |  |
| 25 to <30 | 41 |  | 20.0 (4.40) | |  | 88 | | 20.3 (6.1) |  |
| ≥30 | 20 |  | 17.3 (4.44) | |  | 66 | | 19.5 (5.1) |  |
| **Sleep time** |  |  |  | |  |  | |  |  |
| Tertile 1 | 23 |  | 20.6 (6.3) | |  | 62 | | 19.5 (5.7) |  |
| Tertile 2 | 22 |  | 18.6 (3.4) | |  | 65 | | 20.2 (5.5) |  |
| Tertile 3 | 33 |  | 19.5 (5.2) | |  | 74 | | 19.8 (5.9) |  |
| **Hypertension** |  |  |  | |  |  | |  |  |
| No | 22 |  | 20.2 (6.3) | |  | 65 | | 18.6 (5.5) |  |
| Yes | 57 |  | 19.2 (4.7) | |  | 149 | | 20.3 (5.5) |  |
| **Cardiovascular disease** |  |  |  | |  |  | |  |  |
| No | 73 |  | 19.5 (5.2) | |  | 201 | | 19.8 (5.4) |  |
| Yes | 6 |  | 18.7 (5.6) | |  | 13 | | 19.2 (7.6) |  |
| **Cancer** |  |  |  | |  |  | |  |  |
| No | 73 |  | 19.4 ( 4.6) | |  | 210 | | 19.8 (5.6) |  |
| Yes | 6 |  | 20.4 (10.6) | |  | 4 | | 17.4 (5.4) |  |
| **Diabetes** |  |  |  | |  |  | |  |  |
| No | 54 |  | 19.2 (5.7) | |  | 179 | | 19.6 (5.5) |  |
| Yes | 25 |  | 20.0 (6.2) | |  | 35 | | 20.5 (6.0) |  |
| **Rheumatoid arthritis** |  |  |  | |  |  | |  |  |
| No | 71 |  | 19.4 (5.4) | |  | 182 | | 19.8 (5.5) |  |
| Yes | 8 |  | 20.0 (3.1) | |  | 32 | | 19.4 (5.8) |  |
| **Osteoarthritis** |  |  |  | |  |  | |  |  |
| No | 47 |  | 20.2 (4.9) | |  | 66 | | 19.5 (5.4) |  |
| Yes | 32 |  | 18.5 (5.5) | |  | 148 | | 19.9 (5.6) |  |
| **Chronic respiratory disease** |  |  |  | |  |  | |  |  |
| No | 73 |  | 19.7 (5.3) | |  | 183 | | 20.0 (5.7) |  |
| Yes | 6 |  | 16.4 (2.6) | |  | 31 | | 18.4 (2.6) |  |
| **Mental component of the SF12** |  |  |  | |  |  | |  |  |
| Tertile 1 | 60 |  | 19.2 (5.0) | |  | 146 | | 20.7 (5.6) |  |
| Tertile 2 | 11 |  | 21.9 (4.8) | |  | 37 | | 17.5 (5.5) |  |
| Tertile 3 | 8 |  | 17.9 (6.5) | |  | 31 | | 18.1 (4.5) |  |
| **Platelet count (× 10^9^/L)** |  |  |  | |  |  | |  |  |
| Quartile 1 | 20 |  | 15.7 (4,1) | |  | 33 | | 16.5 (5.0) |  |
| Quartile 2 | 24 |  | 20.3 (5,6) | |  | 53 | | 17.8 (4.9) |  |
| Quartile 3 | 18 |  | 20.1 (4,3) | |  | 62 | | 20.1 (5.3) |  |
| Quartile 4 | 17 |  | 22.0 (4,6) | |  | 66 | | 22.7 (5.1) |  |
| **Platelet volume** |  |  |  | |  |  | |  |  |
| Quartile 1 | 16 |  | 21,1 (6.1) | |  | 66 | | 20.7 (6.1) |  |
| Quartile 2 | 28 |  | 19.2 (4.1) | |  | 52 | | 19.4 (5.8) |  |
| Quartile 3 | 22 |  | 19.3 (6.5) | |  | 46 | | 19.6 (5.0) |  |
| Quartile 4 | 13 |  | 18.2 (3.1) | |  | 50 | | 19.1 (4.9) |  |
|  |  |  |  |  |  | |  |  |  |

MEDAS: Mediterranean Diet Adherence Screener; PA: Physical activity

*P-values were obtained from t-tests or ANOVA

^+^Information available in the subset of participants who wore an accelerometer

**Supplementary Table 2:** Fully-Adjusted Mean Differences in BDNF Levels (ng/mL) in individuals according to different types of CP, and stratified by sex and depressive status

|  |  |  | Non-depressed |  |  | Depressed |  |
| --- | --- | --- | --- | --- | --- | --- | --- |
|  |  | n/total |  |  | n/total |  |  |
| **Musculoskeletal pain (vs no pain)** | **Men** | 110/856 | 1.11 (-0.03; 2.25) | * | 32/72 | -2.87 (-5.42;-0.31) | * |
|  | **Women** | 193/734 | -1.04 (-1.98; -0.10) |  | 111/205 | 0.92 (-0.59; 2.44) |  |
|  | p-int |  | 0.004 | * |  | 0.012 | * |
| **Nociplastic pain (vs no pain)** | **Men** | 6/752 | 4.32 (-0.24; 8.89) | * | 4/44 | -7.02 (-12.6; -1.38) | * |
|  | **Women** | 27/568 | -2.72 (-4.92; -0.52) |  | 34/128 | 0.13 (-2.02; 2.28) |  |
|  | p-int |  | 0.001 | * |  | 0.020 | * |
| **Neuropathic pain (vs no pain)** | **Men** | 9/755 | -1.20 (-4.89; 2.50) | * | 3/43 | -5.71 (-12.1; 0.72) | * |
|  | **Women** | 26/567 | -1.96 (-4.17; 0.25) |  | 16/110 | 1.59 (-1.37; 4.54) |  |
|  | p-int |  | 0.73 | * |  | 0.045 | * |
| **Visceral pain (vs no pain)** | **Men** | 8/754 | -1.63 (-5.54; 2.29) | * | 6/46 | -5.66 (-10.42; -0.91) | * |
|  | **Women** | 22/563 | 0.17 (-2.23; 2.57) |  | 15/109 | -0.58 (-3.64; 2.48) |  |
|  | p-int |  | 0.44 | * |  | 0.075 | * |
| **Vascular pain (vs no pain)** | **Men** | 11/757 | 1.04 (-2.32; 4.39) | * | 8/48 | -3.10 (-7.86; 0.47) | * |
|  | **Women** | 11/552 | -2.18 (-5.55; 1.18) |  | 13/107 | -2.86 (-6.05; 0.33) |  |
|  | p-int |  | 0.184 | * |  | 0.754 | * |

p-int: P value for interaction obtained from Wald tests for interaction terms

**Supplementary Table 3:** Fully-Adjusted Mean Differences in BDNF Levels (ng/mL) in individuals with Severe and Interfering pain compared to their counterparts with Chronic Pain, Stratified by Sex and Depressive Status

|  |  | Non-depressed | |  | Depressed | |
| --- | --- | --- | --- | --- | --- | --- |
|  |  | n | MD (95%CI) |  | n | MD (95%CI) |
| **Intensity (VAS)** |  |  |  |  |  |  |
| Non-severe (≤7) | **Men** | 121 | 1.00 |  | 31 | 1.00 |
| Severe (>7) |  | 16 | 1.82 (-1.36, 5.00) |  | 8 | -2.75 (-7.02, 1.52) |
| Non-severe (≤7) | **Women** | 193 | 1.00 |  | 98 | 1.00 |
| Severe (>7) |  | 22 | -1.72 (-4.43, 0.98) | * | 22 | -0.80 (-3.41, 1.81) |
|  | p-int |  | 0.090 | * |  | 0.454 |
| **Interference in daily living** |  |  |  |  |  |  |
| Non-interfering | **Men** | 110 | 1.00 | * | 24 | 1.00 |
| Interfering |  | 27 | -0.52 (-3.08, 2.04) |  | 15 | -2.37 (-5.89, 1.15) |
| Non-interfering | **Women** | 162 | 1.00 |  |  | 1.00 |
| Interfering |  | 53 | -2.39 (-4.32, -0.45) | * | 71 | -0.77 (-2.83, 1.30) |
|  | p-int |  | 0.016 | * | 49 | 0.44 |

p-int: P value for interaction obtained from Wald tests for interaction terms
